# Triglyceride-glucose index and coronary artery disease: a systematic review and meta-analysis of risk, severity, and prognosis

**DOI:** 10.1186/s12933-023-01906-4

**Published:** 2023-07-06

**Authors:** Shichu Liang, Cui Wang, Jing Zhang, Zhiyue Liu, Yanlin Bai, Zhonglan Chen, He Huang, Yong He

**Affiliations:** 1 Department of Cardiology, West China Hospital, Sichuan University, No.37 GuoXue Alley, Chengdu, 610041 China; 2 Department of Endocrinology & Metabolism, Laboratory of Endocrinology & Metabolism, and Rare Disease Center, West China Hospital, Sichuan University, Chengdu, China; 3 West China School of Nursing, Sichuan University, Chengdu, China; 4 West China School of Medicine, West China Hospital, Sichuan University, Chengdu, Sichuan China

**Keywords:** Triglyceride-glucose index, Insulin resistance, Coronary artery disease, Risk, Severiy, Prognosis, Meta-analysis

## Abstract

**Background:**

The TyG index is an indicator of insulin resistance (IR), which is associated with the development and prognosis of cardiovascular disease. This study aimed to summarize the relationship between the TyG index and the risk, severity, and prognosis of coronary artery disease (CAD) by performing a systematic review and meta-analysis.

**Methods:**

The PubMed, EMBASE, The Cochrane Library, and Web of Science databases were searched for articles published from inception until May 1, 2023. Cross-sectional studies, retrospective or prospective cohort studies recruiting patients with CAD were included. For the analysis of CAD severity, the outcomes were coronary artery calcification, coronary artery stenosis, coronary plaque progression, multi-vessel CAD, and in-stent re-stenosis. For the analysis of CAD prognosis, the primary outcome was major adverse cardiovascular events (MACE).

**Results:**

Forty-one studies were included in this study. Compared to patients with the lowest TyG index, those with the highest TyG index had a higher CAD risk [odds ratio (OR): 1.94, 95% confidence interval (CI) 1.20–3.14, *I*^*2*^ = 91%, *P* = 0.007]. Additionally, these patients were more likely to have stenotic coronary arteries (OR: 3.49, 95% CI 1.71–7.12, *I*^*2*^ = 0%, *P* = 0.0006), progressed plaques (OR: 1.67, 95% CI 1.28–2.19, *I*^*2*^ = 0%, *P* = 0.002), and with more vessels involved (OR: 2.33, 95% CI 1.59–3.42, *I*^*2*^ = 0%, *P* < 0.0001). When calculated as a categorized variable, it appears that acute coronary syndrome (ACS) patients with higher TyG index levels may have a higher incidence rate of MACE [hazard ratio (HR): 2.09, 95% CI 1.68–2.62, *I*^*2*^ = 87%, P < 0.00001], whereas chronic coronary syndrome (CCS) or stable CAD patients with higher TyG index levels showed a trend towards an increased incidence rate of MACE (HR: 1.24, 95% CI 0.96–1.60, *I*^*2*^ = 85%, P = 0.09). When calculated as a continuous variable, ACS patients had an HR of 2.28 per 1-unit/1-standard deviation increment of the TyG index (95% CI 1.44–3.63, *I*^*2*^ = 95%, P = 0.0005). Similarly, CCS or stable CAD patients had an HR of 1.49 per 1-unit/1-standard deviation increment of the TyG index (95% CI 1.21–1.83, *I*^*2*^ = 75%, P = 0.0001). Myocardial infarction with non-obstructive coronary arteries patients had an HR of 1.85 per 1-unit increment of the TyG index (95% CI 1.17–2.93, P = 0.008).

**Conclusions:**

The TyG index is a simple new synthetic index that has been proven to be a valuable tool in the whole-course management of CAD patients. Patients with higher TyG index levels are at a higher risk of CAD, more severe coronary artery lesions, and worse prognosis compared to those with lower TyG index levels.

**Supplementary Information:**

The online version contains supplementary material available at 10.1186/s12933-023-01906-4.

## Background

Cardiovascular diseases are the leading cause of death and disability globally, with coronary artery disease (CAD) being one of the most prevalent cardiovascular disorders. CAD can often lead to acute myocardial infarction (AMI) and ultimately heart failure [1], making early diagnosis and risk stratification essential for determining appropriate clinical management.

Insulin resistance (IR) is now recognized as a novel risk factor for CAD, especially when combined with obesity and dyslipidemia [2]. However, the gold standard for IR, the hypoglycemic-hyperinsulinemic clamp test, is not feasible for large-scale studies due to its time-consuming and labor-intensive nature [3]. Homeostasis model assessment of insulin resistance (HOMA-IR) is a commonly used surrogate indicator but it is expensive and has poor reproducibility [3]. In 2008, the triglyceride-glucose (TyG) index was introduced as a reliable and specific predictor of IR. It has been shown to have a good correlation with the hypoglycemic-hyperinsulinemic clamp test and HOMA-IR [4]. The TyG index is particularly suitable for economically underdeveloped areas where laboratory testing may be inconvenient.

Previous studies have shown that a high TyG index is linked to the development and prognosis of cardiovascular disease (CVD) [5, 6], even in individuals without CAD at baseline [7]. Furthermore, an increasing number of recent studies have reported on the predictive value of the TyG index in CAD. To provide more reliable evidence for clinical practice, a systematic review and meta-analysis were conducted to summarize the relationship between the TyG index, CAD risk, severity, and prognosis.

## Methods

### Study design and literature search

This is a registered meta-analysis on the International Prospective Register of Systematic Reviews (https://www.crd.york.ac.uk/prospero/) with registration number CRD42023422917. The systematic review and meta-analysis included cross-sectional studies, retrospective or prospective cohort studies that recruited patients with CAD, regardless of their nationality, race, age, gender, or course of the disease.

Two authors (S. Liang and J. Zhang) independently searched the PubMed, EMBASE, The Cochrane Library, and Web of Science databases for articles published from inception until May 1, 2023, using the following heading terms: “coronary artery disease”, “coronary heart disease”, “atherosclerotic cardiovascular diseases”, “CAD”, “CHD”, “triglyceride-glucose index”, and “TyG index”. The search was carried out by combining subject words and free words. No language restrictions were used. Relevant literature references were also searched to identify more eligible studies. The literature titles and abstracts were screened for primary screening, and then full-text acquisition and reading of the literature for rescreening were conducted.

### Definitions

The TyG index is calculated as ln[fasting triglycerides (mg/dL) × fasting glucose (mg/dL)/2]. For the analysis of CAD risk and severity, coronary artery calcification (CAC) is defined as coronary artery calcification score (CACS) > 0, and coronary artery stenosis is defined as the maximum intraluminal stenosis in any of the segments of the major epicardial coronary arteries > 70% [8]. Coronary plaque is defined as structures ≥ 1 mm^2^ within or adjacent to the coronary artery lumen, which is characterized from the vessel lumen or surrounding pericardial tissues [8], and plaque progression is defined as the difference of the baseline and follow-up CACS or plaque volume > 0. For the analysis of CAD prognosis, the primary outcome is the major adverse cardiovascular event (MACE), which is defined as the composite outcome of all-cause death, cardiac death, myocardial infarction, revascularization, stroke, and heart failure. The secondary outcomes are all-cause death, cardiac death, myocardial infarction, revascularization, and stroke.

### Data extraction and quality assessment

Two independent readers (S. Liang and C. Wang) extracted the data. They were not blinded to the authors and institutions of included studies. Disagreements were resolved by a third reader (Z. Liu) while Y. He and H. Huang supervised the entire process. This meta-analysis followed the guidelines for the Preferred Reporting Items for Systematic Reviews and Meta-analyses (PRISMA) [9].

The two reviewers independently extracted the following information: the first author, published year, sample size, demographic information, and variables adjusted in multivariate analysis. The risk of bias was assessed using the Newcastle–Ottawa Scale (NOS). The NOS ranges from 0 (lowest) to 9 (highest), and studies with scores ≥ 6 are considered high quality [10].

### Statistical analysis and meta-analysis

The RevMan version 5.3.5 (The Cochrane Collaboration, Copenhagen, Denmark) was properly used in all statistical analyses. Results were compiled using PRISMA. The two authors collecting the data, S. Liang and C. Wang, were not aware of the authors and institutions of included studies.

When the TyG index was analyzed as a categorical variable, the odds ratios (ORs) or hazard ratios (HRs) of patients with the highest TyG index level compared to those with the lowest TyG index level were extracted. When the TyG index was analyzed as a continuous variable, the HRs of the outcome incidence per 1-unit or 1-standard deviation (SD) increment of the TyG index were extracted. Statistical heterogeneity was assessed using the I square test. Heterogeneity was interpreted as absent (*I*^*2*^: 0–25%), low (*I*^*2*^: 25.1–50%), moderate (*I*^*2*^: 50.1–75%), or high (*I*^*2*^: 75.1–100%). A random-effects model was considered when the number of studies was relatively small, and a random-effects model was applied to estimate the continuous outcome data if the *P*-value < 0.1 and an *I*^*2*^ value > 50%, indicating statistical heterogeneity [11]. Otherwise, a fixed-effects model was used. A *P* < 0.05 was regarded as statistical significance for the pooled OR and HR.

## Results

### Literature search and included studies

The authors conducted an online search using databases such as PubMed, EMBASE, The Cochrane Library, and Web of Science. The search initially yielded 381 literature citations, which were reduced to 141 after removing duplicates and irrelevant studies. A review of titles and keywords resulted in the exclusion of 80 studies, leaving 61 abstracts for evaluation by two authors (S. Liang and C. Wang). Thirteen studies were excluded due to their focus on heart failure or arterial stiffness. After full-text evaluation, 41 studies were selected for inclusion in the analysis. The systematic literature search and study selection process is displayed in Additional file 1: Figure S1 using the PRISMA flow chart.

### Quality assessment

The authors assessed the quality of the included studies using the NOS which rates the quality of non-randomized studies based on three criteria: selection, comparability, and outcome. The NOS score of most of the included studies was above 6, indicating good quality. Additional file 1: Table S1 provides the details of the NOS scores for each study.

### The TyG index and CAD risk

**Fig. 1:**
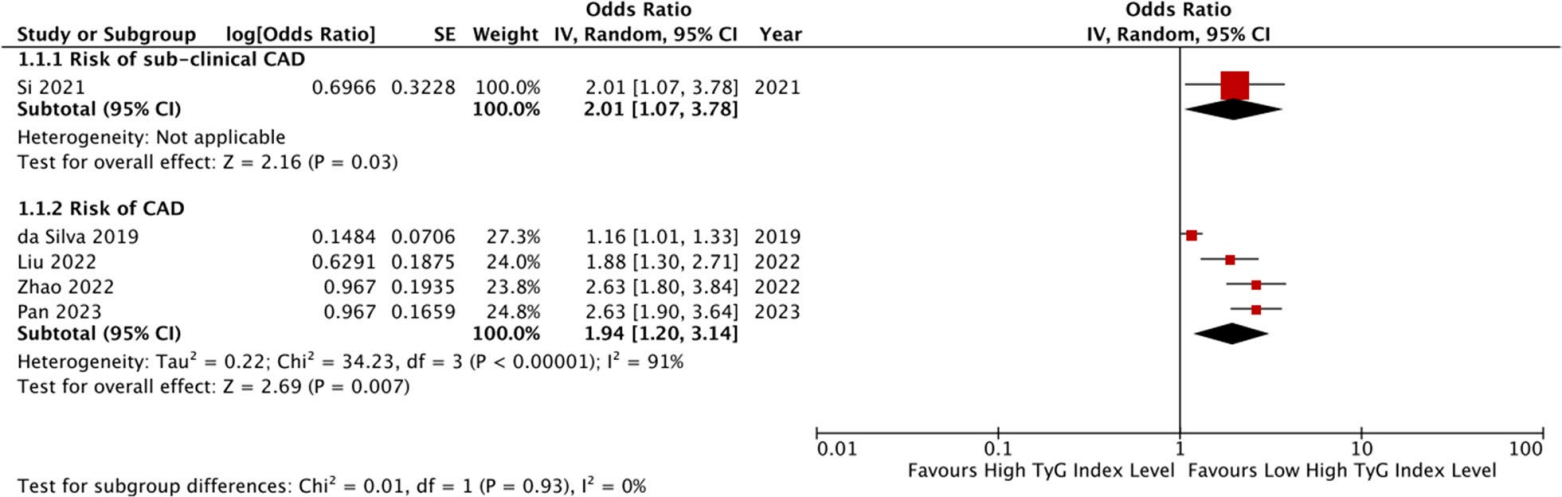
The pooled result of meta-analysis of CAD risk

The authors identified five studies that evaluated the relationship between the TyG index and CAD risk in patients without CAD or with suspected CAD [12–16]. Details of these studies are presented in Table 1. One study [13] found that the TyG index is an independent risk factor for sub-clinical CAD in asymptomatic patients (OR: 2.007, 95% CI 1.066–3.780, *P* = 0.031). The other four studies included postmenopausal women [14], non-alcoholic fatty liver disease (NAFLD) patients [15], hypertensive patients [16], or patients who had at least one CVD in the last 10 years [12]. The pooled results of four studies [12, 14–16] showed that individuals with higher TyG index levels were significantly more likely to have CAD than those with lower TyG index levels (OR: 1.94, 95% CI 1.20–3.14, *I*^*2*^ = 91%, *P* = 0.007, Fig. 1). This finding was consistent when the TyG index was analyzed as a continuous variable in one study [16] (OR per 1-SD increment of the TyG index: 1.49, 95% CI 1.30–1.74, *P* = 0.007).

**Table 1:** Basic information of the included studies for CAD risk and severity

Study	Design	Country	Participants characteristics	Total, n	Mean age, years	Male, n (%)	Diabetes, n (%)	TYGI Analysis	TYGI Cutoff	Variables adjusted in MVA	Outcome OR/HR (95%CI)
CAD risk											
da Silva [12]	PC	Brazil	Patients have at least one CVD in the last 10 years	2330	63.2 ± 8.1	1358 (58.3)	1024 (44.0)	Categorized	NR	Age, sex, use of hypoglycemic, antihypertensive, anticoagulant, lipid-lowing agents, carbohydrate and lipids intake, stroke, peripheral artery disease, and the presence of any other stage of the disease	Risk of CAD: Asymptomatic: 0.98 (0.78–1.17) Symptomatic: 1.16 (1.01–1.33) Treated: 1.03 (0.97–1.10)
Si [13]	RC	China	Asymptomatic patients	697	60 (54, 65)	333 (47.8)	121 (17.6)	Categorized	8.04	Age, sex, smoking, hypertension, DM, LDL-C	Risk of sub-clinical CAD: 2.007 (1.066, 3.780)
Liu [14]	RC	China	Postmenopausal women and suspected CAD	869	NR	NR	225 (25.9)	Categorized	9.432	Age, T2MD, ischemic stroke, SBP, LVEF	Risk of CAD: 1.876 (1.299–2.710)
Zhao [15]	CS	China	NAFLD who underwent coronary angiography	424	NR	266 (62.7)	124 (29.2)	Categorized	9.22	Age, sex, hypertension, DM and smoking history	Risk of CAD: 2.519 (1.559–4.069)
Pan [16]	CS	China	Hypertensive patients without CAD	1841	58.3 ± 14.0	999 (54.3)	480 (26.1)	Categorized and Continuous	8.88	Age, sex, DM, smoking, HDL-C, Hs-CPR, Lp(a), E/e’	Risk of CAD: 2.63 (1.80–3.81)
Coronary artery calcification and stenosis											
Lee [17]	RC	Korea	Asymptomatic adults with T2DM	888	63.9 ± 9.5	523 (58.9)	888 (100.0)	Continuous	NR	Age, sex, HbA1c, duration of DM, SBP, LDL-C, eGFR, UA, smoking, insulin, oral hypoglycemic agents, antiplatelet agents, antihypertensive medication, and statin	Coronary artery stenosis: 3.19 (1.371–7.424)
Kim [18]	PC	Korea	Asymptomatic adults	4319	NR	NR	NR	Continuous	NR	Age, sex, SBP, BMI, LDL-C, HDL-C, smoking, alcohol, and exercise habits	Coronary artery calcification: 1.95 (1.23–3.11)
Thai [19]	CS	France	T2DM	166	58.9 ± 10.8	103 (62.0)	166 (100)	Continuous	10	Duration of DM of 4 years, BMI, eGFR, practicing physical activity, smoking, HbA1c of 7.9% and SBP of 140 mmHg, and logHOMA-IR	Degree of coronary stenosis: 50–69%: 6.89 (1.80–26.44) ≥ 70%: 4.04 (1.00–16.34)
Ding [20]	CS	China	Asymptomatic, non-diabetic patients undergoing maintenance hemodialysis	151	56.66 ± 12.43	84 (55.6)	0 (0)	Continuous	8.82	NR	Coronary artery calcification: 1.281(1.121–1.465)
Coronary plaque											
Park [21]	RC	Korea	Asymptomatic adults	1175	51 ± 7	835 (71.1)	NR	Continuous	NR	Age, sex, BMI, SBP, LDL-C, HDL-C, exercise, alcohol, smoking, presence of DM and hypertension, use of statins and aspirin, and baseline ln(CACS + 1)	Coronary artery calcification progression: 1.82 (1.20–2.77)
Won [22]	PC	Multi-international	Patients underwent CCTA	1143	60.7 ± 9.3	624 (54.6)	319 (27.9)	Continuous	9.03	Age, sex, SBP, BMI, and HDL-C	Coronary plaque progression: 1.083 (1.021–1.150)
Park [23]	RC	Korea	Asymptomatic adults	1250	52.8 ± 6.5	586 (46.9)	0 (0)	Continuous	8.48	Age, sex, SBP, DBP, BMI, LDL-C, HDL-C, and UA	Calcified plaque: 1.488 (0.965–2.295) Non-calcified plaque: 1.581 (1.002–2.493) Mixed plaque: 2.419 (1.051–5.569)
Wang [24]	RC	China	Nondiabetic patients	2719	60.9 ± 6.6	1278 (47.0)	0 (0)	Continuous	9	Age, sex, smoking, alcohol, BMI, eGFR, LDL-C, history of TIA, stroke, CAD, hypertension, dyslipidemia, antihypertensive agents and antiplatelet agents	Presence of coronary plaque: 1.42 (1.14–1.24)
Multi-vessel coronary artery disease											
Thai [19]	CS	France	T2DM	166	58.9 ± 10.8	103 (62.0)	166 (100)	Continuous	10	Duration of DM of 4 years, BMI, eGFR, practicing physical activity, smoking, HbA1c of 7.9% and SBP of 140 mmHg, and logHOMA-IR	Number of vessels with stenosis: 1: 6.88 (1.94–24.38) 2 or 3: 2.74 (0.64–11.76)
Su [25]	RC	China	CAD patients	731	63 (58–68)	429 (58.7)	373 (51.0)	Continuous	7.77	Age, sex, SBP, DBP, BMI, smoking, alcohol, antihypertensive agents, antilipidemic agents, and antiplatelet agents	Multi-vessel coronary artery disease: 2.280 (1.530–3.398)
Wang [26]	RC	China	CAD patients	2792	66 ± 10	1927 (69.0)	1224 (43.8)	Categorized	7.12	Age, sex, BMI, smoking, drinking, hypertension, eGFR, antiplatelet drug use, antilipidemic drug use, and antihypertensive drug use	Multi-vessel coronary artery disease: 1.496 (1.183–1.893)
Xiong [27]	RC	China	ACS patients	1007	66.55 ± 11.41	NR	NR	Continuous	9.18	Age, BMI, hypertension, DM, HR, BNP and SCr	Complexity of CAD: 3.732 (2.330–5.975)
In-stent re-stenosis after drug-eluting stent											
Zhu [28]	RC	China	ACS patients underwent success PCI	1574	58.4 ± 9.4	1218 (77.4)	544 (34.6)	Continuous	9.11	Age, sex, BMI, LVEF, Hs-CRP, hypertension, DM previous PCI, SYNTAX score, target vessel in LAD, target vessel in RCA, the application of intracoronary imagine; DES-sirolimus; stent length, and minimal stent diameter	1.634 (1.125–2.374)
Guo [29]	RC	China	CCS	1414	58.04 ± 0.25	1103 (78)	NR	Continuous	8.6	Age, sex, BMI, previous PCI, presence of PAD, presence of multivessel CAD, Hs-CRP, eGFR, presence of lesion’s length ≥ 20 mm, stent length	1.73 (1.250–2.417)

*ACS* acute coronary syndrome; *BMI* body mass index; *BNP* brain natriuretic peptide; *CABG* coronary artery bypass grafting; *CAD* coronary artery disease; *CACS* coronary artery calcium score; *CCTA* coronary computed tomography angiography; *CCS* chronic coronary syndrome; *CS* cross-sectional study; *DBP* diastolic blood pressure; *DES* drug-eluting stent; *DM* diabetes mellitus; *eGFR* estimated glomerular fltration rate; *HDL-C* high density lipoprotein cholesterol; *HOMA-IR* homeostatic model assessment of insulin resistance; *HR* heart rate; *Hs-CPR* high-sensitivity C-reactive protein; *HbA1c* glycosylated haemoglobin; *LAD* left anterior descending artery; *LDL-C* low density lipoprotein cholesterol; *LVEF* left ventricular ejection fraction; Lp(a) lipoprotein A; *NAFLD* non-alcoholic fatty liver disease; *PAD* peripheral vascular disease; *PC* prospective cohort; *PCI* percutaneous coronary intervention; *RC* retrospective cohort; *RCA* right coronary artery; *SBP* systolic blood pressure; *SCr* serum creatinine; *TIA* transient ischemia attack; *UA* uric acid

### The TyG index and CAD severity

Four studies evaluated the relationship between the TyG index and coronary artery stenosis and/or calcification [17–20], four studies evaluated the relationship between the TyG index and coronary plaque [21–24], four studies evaluated the relationship between the TyG index and multi-vessel CAD [19, 25–27], whilst two studies evaluated the relationship between the TyG index and in-stent re-stenosis (ISR) after drug-eluting stent (DES) [28, 29]. Details of these studies are presented in Table 1. One study [18] found that the TyG index is associated with a higher incidence rate of CAC in asymptomatic adults (OR: 1.95, 95% CI 1.23–3.11, *P* = 0.01). Ding et al. [20] also showed that in asymptomatic, non-diabetic patients undergoing maintenance hemodialysis, the prevalence ratio of CAC was 1.281 (95% CI 1.121–1.465).

**Fig. 2:**
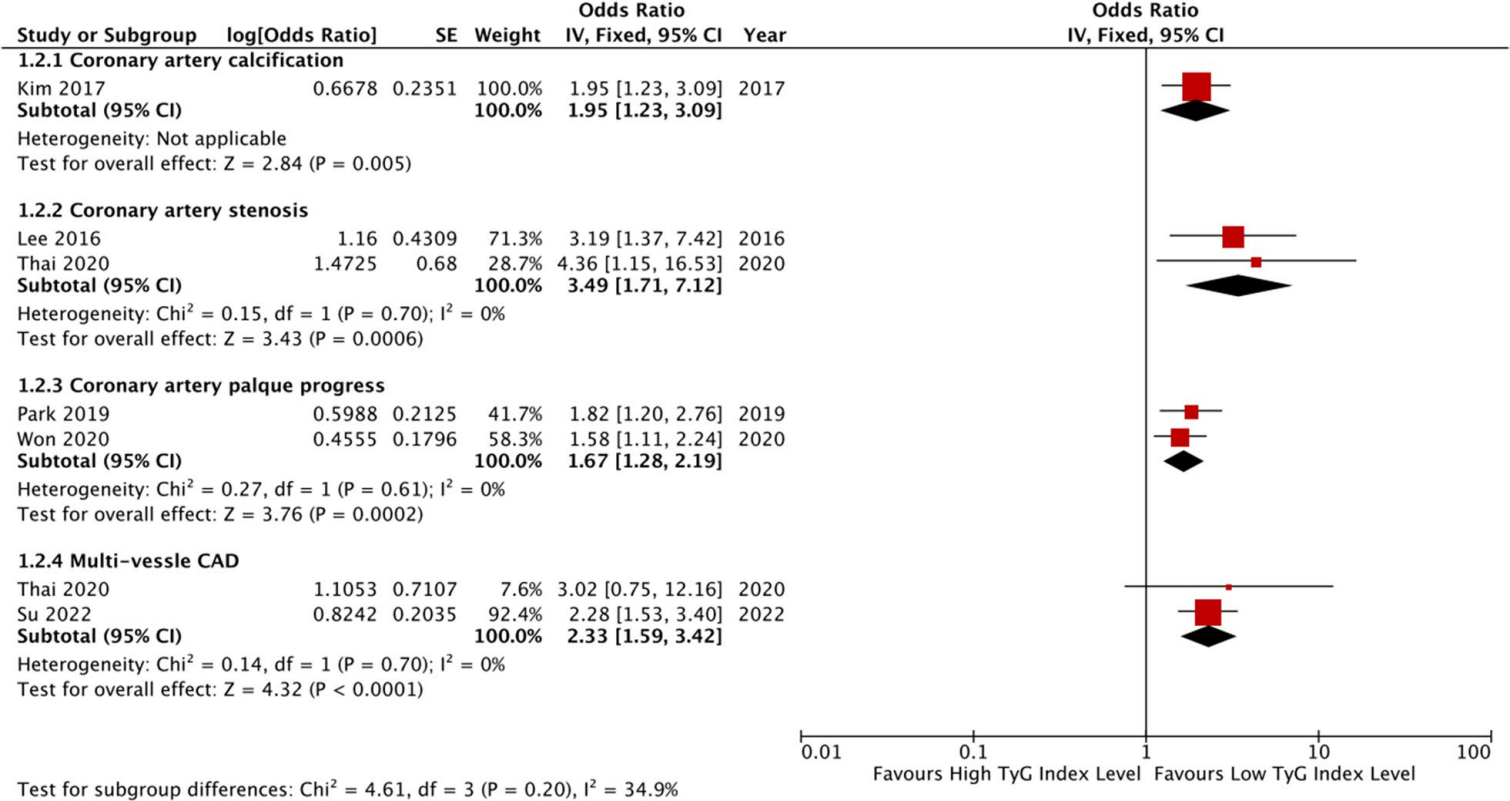
The pooled result of meta-analysis of CAD severity

The pooled results of the studies showed that when the TyG index was analyzed as a continuous variable, higher levels were associated with an increased likelihood of stenotic coronary artery (two studies [17, 19], OR: 3.49, 95% CI 1.71–7.12, *I*^*2*^ = 0%, *P* = 0.0006), progressed plaques (two studies [21, 22], OR: 1.67, 95% CI 1.28–2.19, *I*^*2*^ = 0%, *P* = 0.002), and more vessels involved (two studies [17, 25], OR: 2.33, 95% CI 1.59–3.42, *I*^*2*^ = 0%, *P* < 0.0001). Figure 2 provides details of these results.

### The TyG index and CAD prognosis

In this meta-analysis, a total of fifteen studies evaluated the prognosis of patients with ACS [30–44], seven studies evaluated the prognosis of patients with CCS or stable CAD [29, 45–50], and one study evaluated the prognosis of patients with myocardial infarction with non-obstructive coronary arteries (MINOCA) [51]. The details of the studies are shown in Table 2 and Additional file 1: Table S2.

**Table 2:** Basic information of the included studies for CAD prognosis

Study	Design	Country	Participants characteristics	Total, n	Mean age, years	Male, n (%)	Diabetes, n (%)	TYGI analysis	TYGI Cutoff	Variables adjusted in MVA	Outcome OR/HR (95%CI)	Follow-up duration (months)
ACS												
Mao [30]	CS	China	NSTE-ACS	791	62.5 (53.0–68.0)	295 (67.4)	143 (32.6)	Categorized	8.805	Age, sex, metabolic syndrome, LDL-C, HDL-C, SYNTAX score, CRP, basal insulin, sulfonylurea, metformin, α-glucosidase inhibitor, ACEI/ARB, beta-blocker, and PCI/CABG	①③④⑥⑦	12
Luo [31]	RC	China	STEMI underwent PCI	1092	NR	874 (80.0)	270 (24.7)	Categorized	NR	Age, sex, BMI, SBP, DBP, HR, Killip class > 1, smoking, hypertension, DM, anemia, previous AMI. Atrial fibrillation, BNP, cardiac troponin I, hs-CRP, WBC, neutrophil ratio, platelet, albumin, HbA1c, FPG, TC, triglycerides, HDL-C, LDL-C, UA, eGFR, GPIIb/IIIa antagonists, anticoagulation, lesion vessels, three-vessel disease, Number of stents, Gensini score, LVEF, aspirin, clopidogrel/ticagrelor, statin, beta blockers, ACEI/ARB	①	12
Hu [32]	RC	China	ACS for PCI	9285	59.9 ± 10.05	6996 (75.3)	4074 (43.9)	Categorized	NR	Age, sex, BMI, smoking, hypertension, previous MI, previous stroke, previous PCI, previous CABG, ACS status; non-HDL-C, lipidlowering and antidiabetes agents	①	12
Ma [33]	PC	China	T2DM and ACS for PCI	776	61 ± 10	560 (72.2)	776 (100)	Categorized	9.29	Age, sex, BMI, DBP, HDL-C, HbA1c, smoking, alcohol, presence of PAD, CKD, cardiac failure, previous MI, past PCI, use of insulin and/or oral antidiabetic agents at discharge, CAD severity, presence of lesions > 20 mm long, use of DCB, and complete revascularization	①	30
Zhang [34]	RC	China	ACS for PCI	3181	NR	1409 (75.7)	1231 (38.7)	Categorized	8.88	Age, sex, DM, hypertension, previous AMI, hemoglobin, albumin, eGFR, TGs, LVEF, multi-vessel/LM	①②③④⑤⑥	33.3
Zhao [35]	RC	China	T2DM and NSTE-ACS for PCI	798	60.9 ± 8.3	545 (68.3)	798 (100)	Categorized and Continuous	9.18	Age, sex (female), BMI, SBP, DBP, smoking, drinking, duration of DM, dyslipidemia, prior MI, PCI, stroke and PVD, diagnosis (NSTEMI), TC, HDL-C, eGFR, HbA1c, LVEF, SYNTAX score, LM treatment, DCB use, complete revascularization and number of stents, DAPT at discharge, DAPT interruption in 12 months, statins at discharge, statins interruption in 12 months, oral hypoglycemic agents (metformin, alpha-glucosidase inhibitor, sulfonylurea, dipeptidyl peptidase 4 inhibitor) at discharge and insulin at discharge	①②④⑥	36
Wang [36]	RC	China	DM and ACS for PCI	2531	66.3 ± 6.8	1415 (55.9)	2531 (100)	Categorized and Continuous	9.323	age, male, smoker, previous MI, previous CABG, BMI, AMI, LVEF, left main disease, multi-vessel disease, HbA1c, hs-CRP, statin, insulin	①②⑥⑦	36
Zhao [37]	RC	China	NSTE-ACS for PCI without diabetes	1510	59.7 ± 9.3	1113 (73.7)	0 (0)	Categorized and Continuous	NR	Age, sex, BMI, smoking history, hypertension, dyslipidemia, previous history of MI, PCl, stroke and PAD, NSTE-ACS type, TC, HDL-C, eGFR, HbA1c, LVEF, LM disease, three-vessel disease, CTO, diffuse lesion, in-stent restenosis, SYNTAX score, treatment of LM, LCX, RCA, DES implantation, DCB application, complete revascularization, and number of stents, DAPT at admission, statins at admission, and ACEI/ARB at discharge	①②④⑥⑦	49
Zhang [38]	RC	China	ACS for PCI without diabetes	1655	NR	1223 (73.9)	0 (0)	Categorized	8.33	Age, sex,BMI, SBP, hemoglobin, albumin, Scr, HbAlc, TC, LDL-C, HDL-C, history of smoking and stroke, previous medication history including beta-blocker and statins, and statins treatment during hospitalization	①②③④⑤⑥⑦	35.6
Jiao [39]	RC	China	ACS for PCI over 80	662	81.87 ± 2.14	476 (71.9)	231 (34.9)	Categorized and Continuous	NR	Age, sex, BMI, SBP, DBP, LVEF, Gensini score, hypertension, DM, hyperlipidemia, previous MI, previous stroke, CKD, smoking, TC, LDL-C, HDL-C, eGFR, UA, aspirin, clopidogrel, statin, β-blocker, ACEI/ARB, LM lesion, multivessel lesion and treatment	①②	63
Karadeniz [40]	RC	Turkey	ACS for PCI	1694	64.0 ± 14.4	1191 (70.3)	489 (28.9)	Categorized	9.3/9.2	Age, neutrophil, lymphocyte, CRP	①	60
Guo [41]	RC	China	ACS with prediabetes for PCI	2300	58.87 ± 10.27	1505 (74.1)	0 (0)	Categorized and Continuous	8.83	Age, sex, BMI, SBP, DBP, smoking, hypertension, hyperlipemia, LDL-C. HDL-C, Scr, eGFR, BNP, CRP	①③④⑥⑦	18
Qin [42]	RC	China	T2DM and ACS for PCI	899	NR	NR	899 (100)	Categorized	NR	Duration of DM, TG, FBG, SCr, WBC, neutrophil, fibrinogen, LVEF, GRACE score	①	23
Pang [43]	RC	China	NSTE-ACS for PCI	515	62.3 ± 10.1	361 (70.1)	189 (36.7)	Categorized	NR	LVEF, GRACE score, multivessel disease, previous PCI	①	24
Shen [44]	RC	China	DM and ACS	231	81.58 ± 1.93	156 (67.5)	231 (100)	Categorized	NR	Age, sex, BMI, SBP, DBP, LVEF, Gensini score, hypertension, hyperlipidemia, previous MI, previous stroke, CKD, current smoking, TC, LDL-C, HDL-C, eGFR, UA, aspirin, clopidogrel, statin, β-blocker, ACEI/ARB, LM lesion, multivessel lesion and treatment	②	49
CCS and stable CAD												
Jin [45]	CC	China	T2DM and CAD	800	NR	560 (70.0)	800 (100)	Categorized and Continuous	9.16	Age, sex, BMI, hypertension, family history of CAD, smoke, HDL-C, non-HDL-C, SCr, UA, hsCRP and Gensini score	①	36
Jin [46]	CC	China	T2DM and CAD	1740	NR	1254 (72.1)	468 (37.3)	Continuous	9.17	BMI, LVEF, hypertension, DM, UA, smoking, hsCRP, HDL-C and LDL-C	①	36
Neglia [47]	RC	Italy	CCS	1097	72 (64–77)	821 (75.0)	430 (39.0)	Categorized	9.22	Sex, previous MI and/or coronary revascularization, LDL-C, obstructive CAD, SSS > 7 and Hs-CRP	②③⑥	
Yang [48]	PC	China	CCS without diabetes	5489	NR	NR	0 (0)	Continuous	8.92	NR	①	29
Chen [49]	RC	China	CAD and DM underwent OPCABG	1578	62.9 ± 8.0	1116 (70.7)	1578 (100)	Categorized and Continuous	NR	Age, sex, BMI, current smoking, hypertension, previous MI, previous stroke, past PCI, cardiac failure, CKD, preoperative LVEF, insulin dependence, LDL-c, HDL-C, diagnosis, extent of CAD, left main disease, complete revascularization, and use of IABP	①②⑥⑦	24
Guo [29]	RC	China	CCS	1414	58.04 ± 0.25	1103 (78.0)	NR	Continuous	8.83	Age, sex, BMI, previous PCI, presence of PAD, presence of multivessel CAD, Hs-CRP, eGFR, presence of lesion’s length ≥ 20 mm, stent length	④	60
Lin [50]	RC	China	CTO with T2DM	681	59.16 ± 9.82	563 (82.7)	681 (100)	Categorized	9.02	Age, BMI, SBP, previous MI, previous PCI, TC, LDL-C, TG, FPG, eGFR, UA, isulin	①④⑥	22
MINOCA												
Gao [51]	PC	China	MINOCA	1179	NR	NR	NR	Continuous	8.99	Age, sex, MI type, hypertension, DM and dyslipidemia	①	41.7

① MACE; ② All-cause death; ③ Cardiac death; ④ Revasculation; ⑤ Cardiac rehospitalization; ⑥ MI; ⑦ Stroke

*ACS* acute coronary syndrome; *BMI* body mass index; *BNP* brain natriuretic peptide; *CABG* coronary artery bypass grafting; *CAD* coronary artery disease; *CACS* coronary artery calcium score; *CCTA* coronary computed tomography angiography; *CKD* chronic kidney disease; *CS* cross-sectional study; *CTO* chronic total occlusion; *CCS* chronic coronary syndrome; *DBP* diastolic blood pressure; *DES* drug-eluting stent; *DCB* drug-coated balloon; *DM* diabetes mellitus; *eGFR* estimated glomerular fltration rate; *HDL-C* high density lipoprotein cholesterol; *HOMA-IR* homeostatic model assessment of insulin resistance; *HR* heart rate; *Hs-CPR* high-sensitivity C-reactive protein; *HbA1c* glycosylated haemoglobin; *LAD* left anterior descending artery; *LDL-C* low density lipoprotein cholesterol; *LM* left main stem; *LVEF* left ventricular ejection fraction; *Lp(a)* lipoprotein A; *MI* myocardial infarction; *NAFLD* non-alcoholic fatty liver disease; *PAD* peripheral vascular disease; *PC* prospective cohort; *PCI* percutaneous coronary intervention; *RC* retrospective cohort; *RCA* right coronary artery; *SBP* systolic blood pressure; *SCr* serum creatinine; *SSS* summed stress score; *TC* total cholesterol; *TIA* transient ischemia attack; *UA* uric acid

**Fig. 3:**
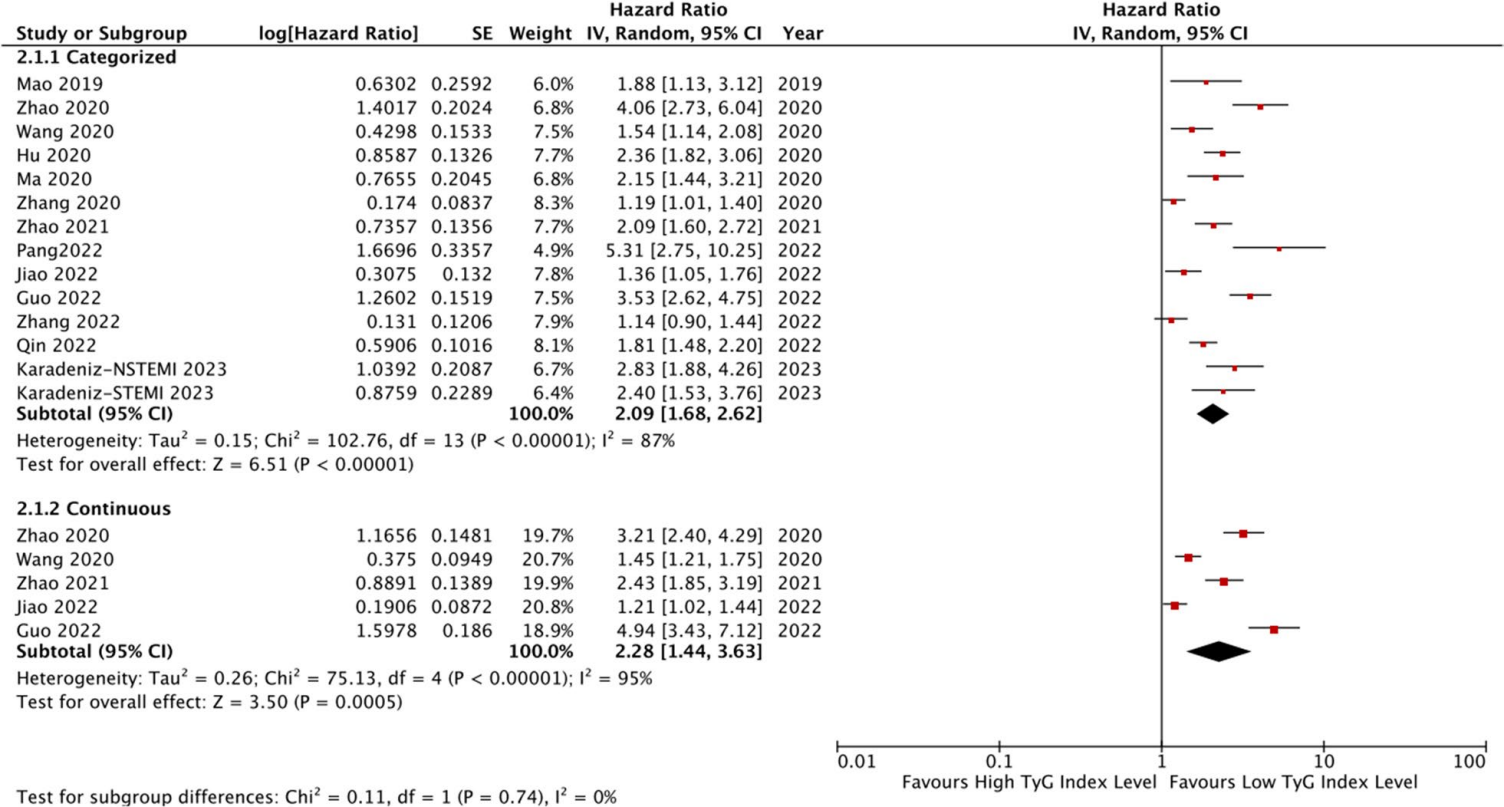
The pooled result of the incidence rate of MACE in ACS patients

The meta-analysis of thirteen studies [30, 32–42, 44] revealed that ACS patients with the highest TyG index level had a significantly increased incidence rate of MACE compared to those with the lowest level (HR: 2.09, 95% CI 1.68–2.62, *I*^*2*^ = 87%, *P* < 0.00001). This association was also observed when the TyG index was analyzed as a continuous variable using five studies [35–37, 39, 41], with an HR per 1-unit/1-SD increment of the TyG index of 2.28 (95% CI 1.44–3.63, *I*^*2*^ = 95%, *P* = 0.0005). Figure 3 provides more details on these findings.

**Fig. 4:**
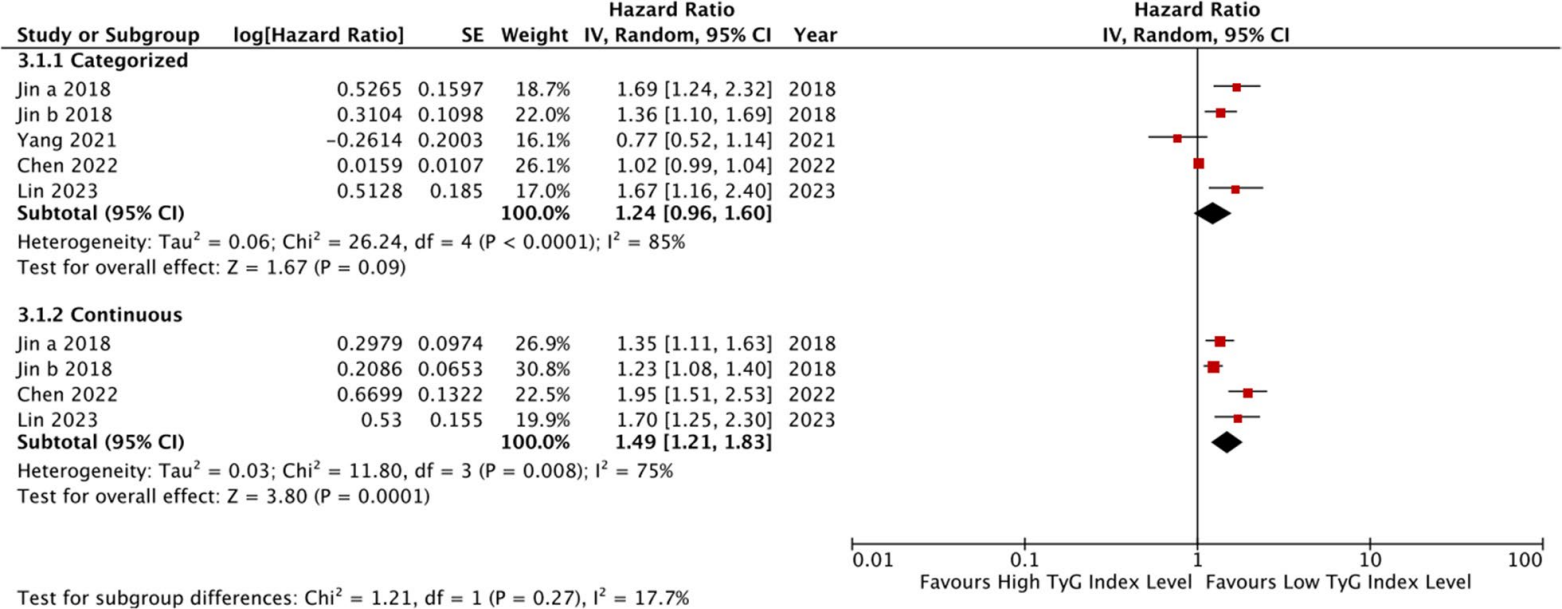
The pooled result of the incidence rate of MACE in CCS and stable CAD patients

**Fig. 5:**
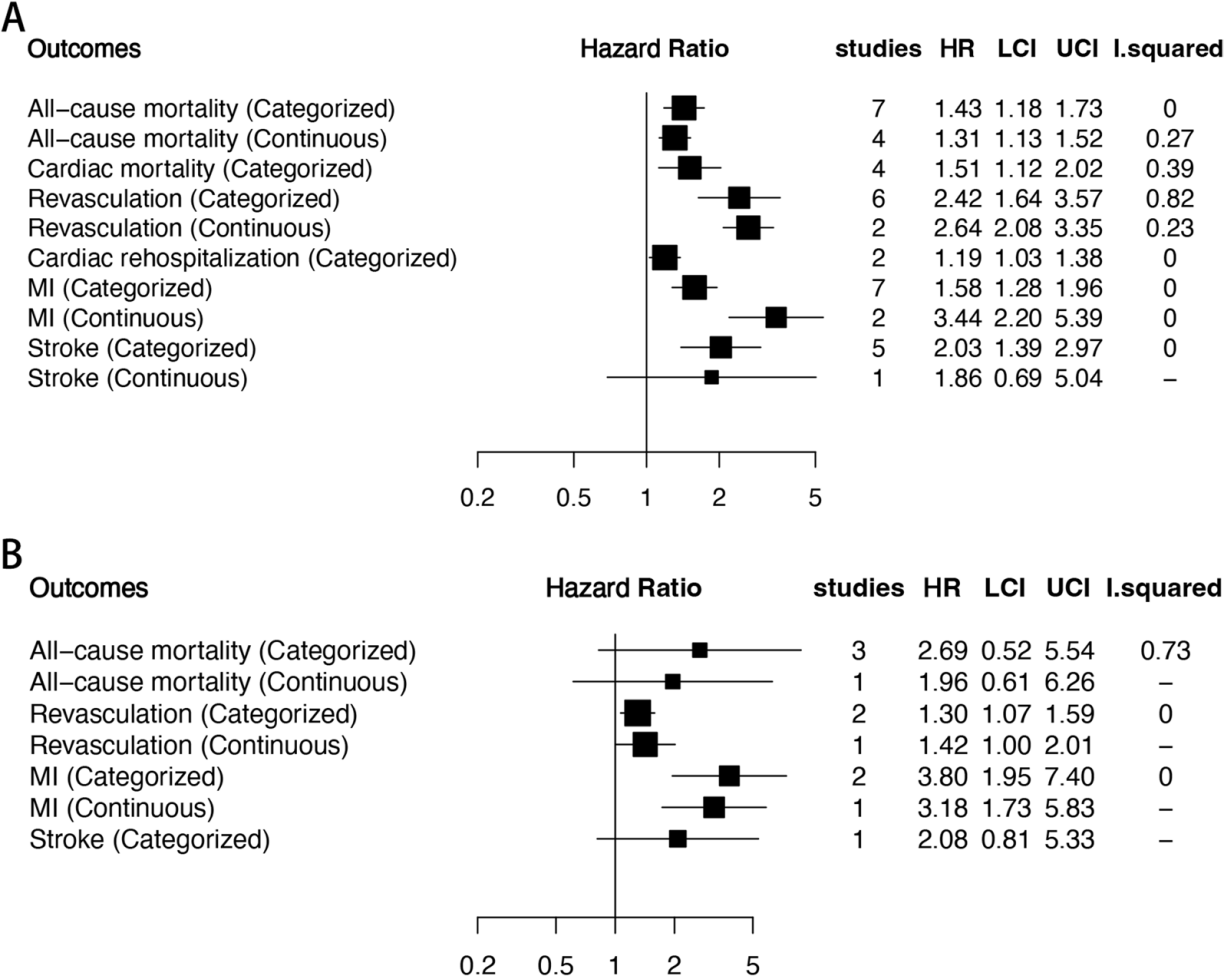
The pooled result of the incidence rate of the secondary outcomes (A.ACS patients; B.CCS and stable CAD patients)

Five studies [45, 46, 48–50] indicated that individuals with the highest TyG index level have a higher incidence rate of MACE compared to those with the lowest level among CCS or stable CAD patients, although the trend was not statistically significant (HR: 1.24, 95% CI 0.96–1.60, *I*^*2*^ = 85%, P = 0.09). Four studies analyzed the TyG index as a continuous variable [45, 46, 49, 50], and found that the risk of MACE increased by 1.49 times per 1-unit/1-SD increment of the TyG index (95% CI 1.21–1.83, *I*^*2*^ = 75%, *P* = 0.0001) (Fig. 4). Only one study [51] analyzed the TyG index as a continuous variable, and reported an HR of 1.85 per 1-unit increment of the TyG index (95% CI 1.17–2.93, *P* = 0.008). Additional file 1: Table S3 and Figure 5 present the results of the secondary outcomes.

### Publication bias

Additional file 1: Figure S2 shows the funnel plots for the association between the TyG index and the incidence rate of MACEs in ACS patients. The plots appear symmetrical upon visual inspection, indicating a low risk of publication bias. However, due to a limited number of datasets, it was challenging to estimate publication bias for the meta-analysis of other outcomes and diseases such as CCS and MINOCA.

## Discussion

### Main finding

**Fig. 6:**
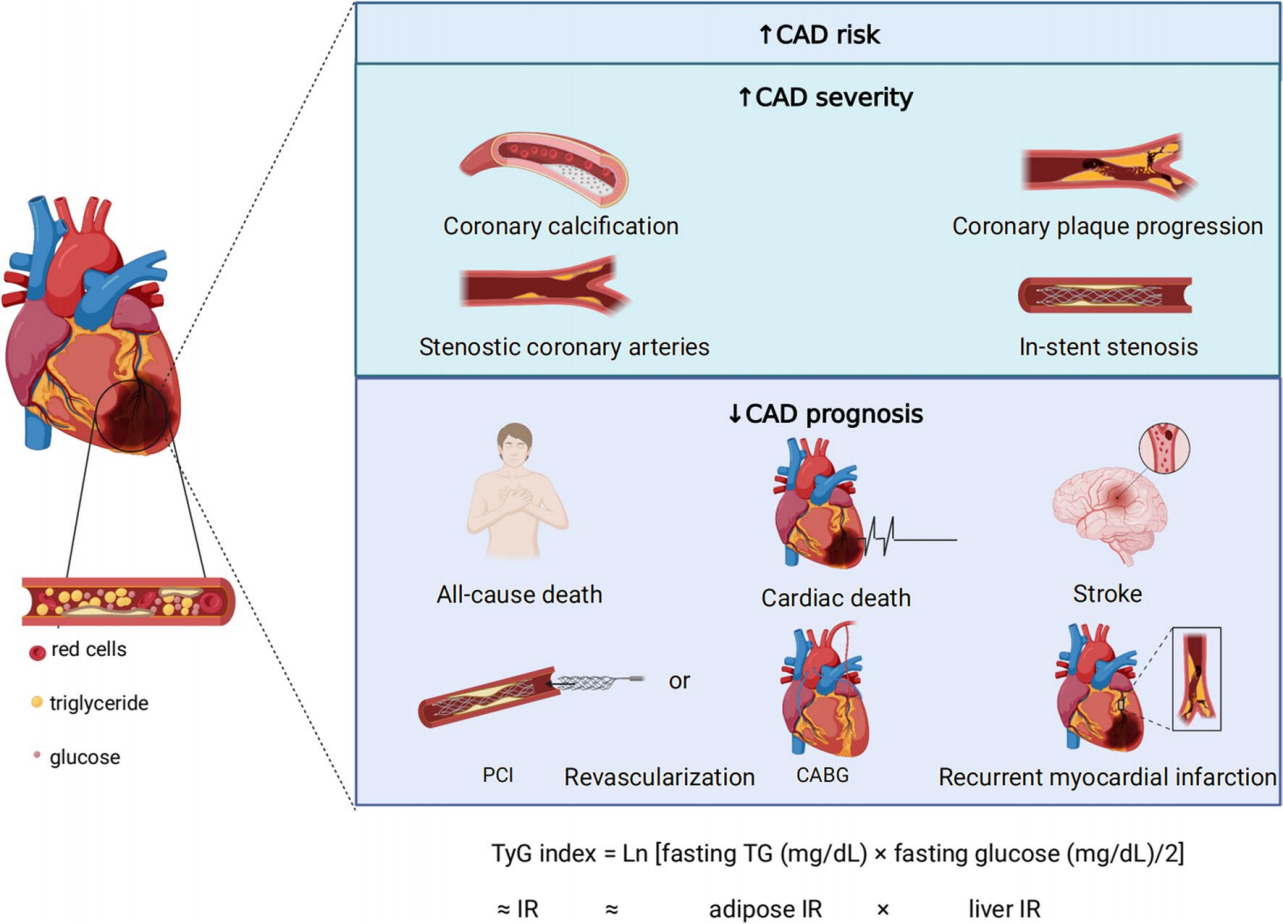
The summary of the study (*CABG* coronary artery bypass grafting; *IR* insulin resistance; *PCI* percutaneous coronary intervention)

The TyG index is a composite index that combines both fasting triglyceride levels and fasting blood glucose levels. It is a highly sensitive and specific marker for identifying IR, which is a risk factor for cardiovascular events [52]. An increased baseline TyG index can help identify individuals who are at high risk for these events. This meta-analysis found that individuals with the highest TyG index have a greater risk of coronary artery disease, more severe coronary artery lesions, and a worse prognosis compared to those with the lowest TyG index (Fig. 6**)**.

### Higher TyG index is associated with higher CAD risk

IR may precede the onset of diabetes and CVDs. Even apparently healthy adults may still have IR, which is a key factor in the development of atherosclerosis [55]. In healthy individuals, IR can lead to abnormal metabolism and promote the development of CAD. A previous meta-analysis suggested that a higher TyG index may serve as an independent predictive indicator for an increased risk of CAD incidence in individuals without pre-existing CAD [7]. IR may also serve as a latent risk factor for the interactions between glucose intolerance and CAD [53]. Under normal physiological conditions, insulin induces vasodilation actions by producing nitric oxide. However, dysregulated insulin signaling can harm bioavailable nitric oxide and contribute to vascular stiffening [54]. Additionally, in patients receiving coronary artery bypass graft (CABG) surgery, a higher TyG index is proven to be associated with symptomatic graft failure due to endothelial dysfunction caused by the proinflammatory and procoagulatory effect of IR [49]. Our study contributes additional evidence on the association between the TyG index and the risk of CAD, which indicated that individuals with a higher TyG index have an increased susceptibility to developing CAD.

### Higher TyG index is associated with more severe coronary lesions

Nonenzymatic glycosylation of lipids is one of the factors that contributes to atherosclerosis, along with hypertriglyceridemia and hyperglycemia. In particular, hypertriglyceridemia can lead to the formation of low-density lipoproteins, which can promote atherosclerosis and weaken the protective effect of high-density lipoproteins [56]. What is more, hyperglycemia can be directly and/or indirectly related to the acceleration of atherosclerosis [57]. The severity of CAD is strongly associated with the extent of arterial stenosis and calcification, the presence of coronary plaque, the occurrence of multi-vessel lesions, and the development of ISR following the placement of DES. In a study by Thai et al. [19], a TyG index above 10 was significantly associated with coronary stenosis above 70%, with a sensitivity of 57% and specificity of 75%. Research has shown that the TyG index, compared with HOMA-IR, is more independently associated with the presence of calcified coronary artery plaques in healthy Korean adults undergoing cardiac computed tomography [18], even in those without traditional CVD risk factors [23]. Higher TyG index was also found to be associated with an increased presence of non-calcified and mixed coronary artery laques [23]. Multiple vessel lesions are strongly associated with a poor prognosis in CAD and can increase the complexity of PCI procedures. Wang et al. has demonstrated a dose–response relationship between the TyG index and the risk of developing multiple vessel CAD, with this association being particularly significant in males and older individuals [26]. ISR poses a significant challenge following PCI, and there is an independent positive correlation between an elevated TyG index and DES-ISR. However, the additional predictive value provided by the TyG index is minimal [28]. Therefore, current guidelines recommend evaluating the 10-year CVD risk in apparently healthy adults [58, 59], and the TyG index can provide new insight for identifying the development of CVDs at an early stage.

### Higher TyG index is associated with poorer prognosis

Compared to CAD patients without metabolic syndrome (MeS), those with MeS tend to have more severe coronary lesions and a higher incidence rate of MACE [60]. Even in patients with MINOCA, the presence of MeS significantly increases the risk of MACE [61]. In a retrospective cohort study of critically ill patients with CAD, a higher TyG index was found to be associated with greater mortality, as well as increased length of stay in the intensive care unit (ICU) and hospital, which imposes a significant financial burden on both families and society [62]. Consistent with previous studies, our meta-analysis found that ACS, CCS, and MINOCA patients with higher TyG index have an increased incidence rate of MACE, indicating that higher TyG index is associated with poorer outcomes in CAD.

The relationship between the TyG index and poor prognosis in CAD patients may be due to the association between IR and CAD. The TyG index reflects IR from both fasting blood glucose, which indicates IR from the liver, and fasting triglycerides, which indicate IR from adipose cells [63]. Therefore, the TyG index provides a more comprehensive evaluation of IR. IR can lead to an increase in sympathetic nervous system activity and renal sodium retention, along with hyperinsulinemia, resulting in increased blood pressure and elevated heart burden. Additionally, the metabolic effects of IR, such as hyperglycemia and dyslipidemia, have a synergistic effect with elevated blood pressure, leading to damage to the vascular and kidney systems, which can easily cause damage to the renal and cardiovascular systems [64]. Moreover, IR and insufficient insulin signaling can cause inappropriate activation of the renin–angiotensin–aldosterone system, oxidative stress, inflammation, and dysfunctional immune modulation, all of which can compromise cardiac function [65, 66]. For patients without diabetes, IR has also been found to be associated with reduced cardiac autonomic function, particularly vagal activity [67]. Furthermore, Gao et al. [68] found that in patients with chronic total occlusion lesions, an increased TyG index was strongly associated with less developed collateralization, indicating that these patients may face a larger ischemic area.

### TyG index has incremental value in CAD risk, severity and prognosis

The baseline TyG index can help identify individuals at a higher risk of cardiovascular events at an early stage. In comparison to other indices such as triglycerides, atherogenic index of plasma, triglycerides to high-density lipoprotein cholesterol ratio, and lipoprotein combine index-adjusted HR, the TyG index has shown the best ability to predict MACE [52]. In clinical practice, different scoring systems are commonly used to manage patients with CAD throughout their treatment. The TyG index has shown to have independent and commendable performance in various aspects related to CAD. Therefore, it is possible that combining the TyG index with these scoring systems may provide additional value in managing CAD patients [69, 70].

The Framingham risk score (FRS) is a tool used to assess the risk of CVD in individuals. It takes into account factors such as gender, age, total cholesterol, high-density lipoprotein, systolic blood pressure level, and smoking status to stratify individuals based on their risk level. Guo et al. [71] found that the TyG index is significantly correlated with intermediate- or high-risk patients for CVD, suggesting that the TyG index can be used as an additional factor in assessing CVD risk. Sánchez-Íñigo et al. [52] included the TyG index in the FRS, and found the predictive accuracy of FRS was improved, especially in patients with intermediate risk (10–20% risk). In a 16-year follow-up study by Barzegar et al. [72], the TyG-index was found to be significantly associated with the risk of CVD/CHD incidence, especially among individuals younger than 60 years old. However, adding the TyG-index to the FRS did not provide better predictive ability for CVD risk. The differences of the population may be one possible explanation for the variations in different research findings.

The TyG index can also be applied to other scoring systems. The Gensini score and the SYNTAX score reflect the burden of plaque, the type and complexity of plaques, respectively. In our included studies, Shen et al. [44] found that incorporating the TyG index into the prediction model, along with left ventricular ejection fraction (LVEF) and Gensini score, significantly improves the ability for predicting the risk of all-cause mortality. In addition, Xiong et al. [73] also found that adding the residual SYNTAX score and TyG index to the baseline risk model had an incremental impact on the prediction of MACE. The GRACE score is used to predict in-hospital mortality of patients with ACS as well as the 6-month all-cause mortality rate after discharge. In our meta-analysis, Qin et al. [42] and Pang et al. [43] found that combining TyG index and the GRACE score can provide a better predictive value for the clinical prognosis of ACS patients.

### Current stage and future prospects

In patients with ACS and CCS without diabetes [38, 48], the TyG index has been identified as an independent factor for those with well-controlled cholesterol levels. This indicates that the TyG index can be used as a tool for risk stratification and prognosis assessment in clinical practice, allowing for a comprehensive evaluation of metabolic status and cardiovascular risk in CAD patients. This tool can be utilized for clinical management and intervention strategies for CAD patients. However, it is important to note that the application of the TyG index in CCS patients may differ slightly from that in ACS patients. The value of the TyG index in ACS patients may be influenced by stress hyperglycemia [74], which can exacerbate the index and not reflect the true metabolic status of the patients. Additionally, ACS patients are more likely to experience a poor prognosis, which can obscure the prognostic effect of the TyG index.

What is more, one concerns have been raised regarding the use of baseline fasting triglycerides and fasting blood glucose as predictors for the prognosis of CAD in most studies. This is because CAD is a dynamic and progressive disorder, which makes it uncertain whether the TyG index based on a specific situation can accurately predict its progression [75]. To address this issue, Wang et al. monitored changes in the TyG index during the follow-up period and found that individuals with larger fluctuations in the TyG index were more likely to have a higher cardiovascular risk [76]. Similarly, Cui et al. proposed the use of the cumulative TyG index, which is calculated by summing up the average TyG index for each pair of consecutive examinations multiplied by the time between these two visits in years. They found that the cumulative TyG index was a better predictor of cardiovascular prognosis than the TyG index at baseline [6]. Therefore, dynamic monitoring of the TyG index during the follow-up duration may be a more effective approach to achieving good whole-course management in CAD patients [77].

It is also worth noting that an elevated level of triglycerides is linked to CVD and is a crucial factor in residual risk after statin therapy [78]. While epidemiological studies have found a correlation between triglycerides and cardiovascular risk, almost all clinical studies that have intervened with triglycerides have not yielded positive results [78]. The TyG index obtained by combining fasting glucose and triglycerides levels can provide more information. However, further research is needed to investigate whether drug intervention with the TyG index can improve prognosis.

## Strength and limitations

Our study represents the first registered systematic review and meta-analysis to comprehensively summarize the studies of the TyG index and CAD, providing an evidence-based medicine basis for clinical practice. We found that the TyG index is a significant indicator for CAD risk prediction, severity assessment, and prognosis evaluation. Our pooled ORs and HRs were obtained after multivariate analyses or propensity score matching, which accounted for a variety of confounders. However, our study has some limitations. As a meta-analysis of cohort and cross-sectional studies, we cannot determine a cause-and-effect association, limiting the strength of evidence. Additionally, despite controlling for several confounders, even some studies considered exercising habits [18, 19], residual factors such as dietary habits and lifestyles cannot be eliminated [5]. Also, the definition of the endpoint might be slightly different, such as the target vessel revascularization might be better reflect the prognosis of the patients, whilst the ischemic-driven revascularization might also include non-target vessels, which was affected by the willingness of the patients and medical reimbursement [48]. Furthermore, differences in the definition of MACEs may affect the interpretation of the results. Most of the data analyzed were obtained from Asian populations, and more research is needed to determine the applicability of the TyG index in other races. Despite these limitations, our study suggests that the TyG index is a meaningful indicator in the management of CAD patients.

## Conclusions

The TyG index is a straightforward and effective synthetic index that has been shown to be a valuable indicator for predicting the risk of CAD, assessing its severity, and evaluating prognosis. Individuals with a higher TyG index are more likely to face an increased risk of CAD, more severe coronary artery lesions, and a poorer prognosis compared to those with a lower TyG index. In the whole-course management of CAD patients, monitoring changes in the TyG index over time may be helpful in ensuring comprehensive and effective treatment.

## Supplementary Information

**Additional file 1: Table S1.** NOS of the included studies. **Table S2.** Definition of the primary endpoint. **Table S3.** Results of the secondary outcomes. **Figure S1.** The flow chart of the process (*83 from PubMed, 94 from EMbase, 92 from The Cochrane Library and 112 from Web of Science). **Figure S2.** Results of the secondary outcomes.

## Data Availability

All data generated or analysed during this study are included in this published article.

## Abbreviations

ACS: Acute coronary syndrome
AMI: Acute myocardial infarction
CACS: Coronary artery calcification score
CABG: Coronary artery bypass graft
CAD: Coronary artery disease
DES: Drug-eluting stent
HOMA-IR: Homeostasis model assessment of insulin resistance
HR: Hazard ratios
IR: Insulin resistance
ISR: In-stent re-stenosis
MACE: Major adverse cardiovascular event
MeS: Metabolic syndrome
MINOCA: Myocardial infarction with nonobstructive coronary arteries
NOS: Newcastle–Ottawa Scale
TyG: Triglyceride-glucose
OR: Odds ratio
