# Supplementary material for: Triglyceride-glucose index and coronary artery disease: a systematic review and meta-analysis of risk, severity, and prognosis
Source: Cardiovasc Diabetol. 2023 Jul 6;22:170. doi: 10.1186/s12933-023-01906-4 (PMC10327356; doi:10.1186/s12933-023-01906-4)
Supplement: Supplementary file 1 — Additional file 1: Table S1. NOS of the included studies. Table S2. Definition of the primary endpoint. Table S3. Results of the secondary outcomes. Figure S1. The flow chart of the process (*83 from PubMed, 94 from EMbase, 92 from The Cochrane Library and 112 from Web of Science). Figure S2. Results of the secondary outcomes. [file 12933_2023_1906_MOESM1_ESM.docx]

**Additional file**

**Search strategies**

1.Embase（94）

#1 'coronary artery disease':ab,ti OR 'coronary heart disease':ab,ti OR 'atherosclerotic cardiovascular diseases':ab,ti OR 'CAD':ab,ti OR 'CHD':ab,ti OR 'ASCVD':ab,ti

#2 'triglyceride glucose index':ab,ti OR 'tyg index':ab,ti

#1 AND #2

2.PubMed （83）

("triglyceride glucose index"[Title/Abstract] OR "tyg index"[Title/Abstract]) AND ("Coronary Artery Disease"[MeSH Terms] OR "Coronary Disease"[MeSH Terms] OR ("Coronary Artery Disease"[Title/Abstract] OR "coronary heart disease"[Title/Abstract] OR "atherosclerotic cardiovascular diseases"[Title/Abstract] OR "CAD"[Title/Abstract] OR "CHD"[Title/Abstract] OR "ASCVD"[Title/Abstract]))

3.Cochrane（92）

#1 MeSH descriptor: [Coronary Artery Disease] explode all trees

#2 MeSH descriptor: [Coronary Disease] explode all trees

#3 (coronary artery disease):ti,ab,kw OR (coronary heart disease):ti,ab,kw OR (atherosclerotic cardiovascular diseases):ti,ab,kw OR (CAD):ti,ab,kw OR (CHD):ti,ab,kw

#4 (ASCVD):ti,ab,kw

#5 #1 OR #2 OR #3 OR #4

#6 (triglyceride glucose index):ti,ab,kw OR (tyg index):ti,ab,kw

#7 #5 AND #6

4.Web of science（112）

#1 TS=("coronary artery disease") OR TS=("coronary heart disease") OR TS=("atherosclerotic cardiovascular diseases") OR TS=(CAD) OR TS=(CHD) OR TS=(ascvd)

#2 (TS=("triglyceride glucose index")) OR TS=("tyg index")

#3 #1 AND #2

**Table S1. NOS of the included studies.**

| **Study** | **Selection** | | | | **Comparability** | **Outcome** | | | **Overall** |
| --- | --- | --- | --- | --- | --- | --- | --- | --- | --- |
|  | **1** | **2** | **3** | **4** | **1** | **1** | **2** | **3** |  |
| **da Silva2019 [12]** | 0 | 0 | 1 | 0 | 2 | 1 | 0 | 0 | 4 |
| **Si2021 [13]** | 0 | 0 | 1 | 0 | 2 | 1 | 0 | 0 | 4 |
| **Liu2022 [14]** | 0 | 0 | 1 | 0 | 2 | 1 | 0 | 0 | 4 |
| **Zhao2022 [15]** | 0 | 1 | 1 | 0 | 2 | 1 | 0 | 0 | 5 |
| **Pan2023 [16]** | 1 | 1 | 1 | 1 | 2 | 1 | 0 | 0 | 7 |
| **Lee2016 [17]** | 0 | 1 | 1 | 0 | 2 | 1 | 0 | 0 | 5 |
| **Kim2017 [18]** | 1 | 1 | 1 | 1 | 2 | 1 | 0 | 0 | 7 |
| **Thai2020 [19]** | 0 | 1 | 1 | 1 | 2 | 1 | 0 | 0 | 6 |
| **Ding2023 [20]** | 0 | 0 | 1 | 1 | 2 | 1 | 0 | 0 | 5 |
| **Park2019 [21]** | 1 | 1 | 1 | 1 | 2 | 1 | 1 | 1 | 9 |
| **Won2020 [22**] | 0 | 1 | 1 | 1 | 2 | 1 | 1 | 1 | 8 |
| **Park2020 [23]** | 1 | 1 | 1 | 1 | 2 | 1 | 0 | 0 | 7 |
| **Wang2022 [24]** | 1 | 1 | 1 | 1 | 2 | 1 | 0 | 0 | 7 |
| **Su2022 [25]** | 0 | 1 | 1 | 0 | 2 | 1 | 0 | 0 | 5 |
| **Wang2022[26]** | 0 | 0 | 1 | 1 | 2 | 1 | 1 | 1 | 7 |
| **Xiong2023 [27]** | 0 | 1 | 1 | 0 | 2 | 1 | 0 | 0 | 5 |
| **Zhu2021 [28]** | 0 | 1 | 1 | 1 | 2 | 1 | 1 | 1 | 8 |
| **Guo2023 [29]** | 0 | 1 | 1 | 0 | 2 | 1 | 1 | 1 | 7 |
| Mao2019 [30] | 0 | 1 | 1 | 1 | 2 | 1 | 1 | 1 | 8 |
| **Luo2019 [31]** | 0 | 1 | 1 | 1 | 2 | 1 | 1 | 1 | 8 |
| **Hu2020 [32]** | 0 | 1 | 1 | 1 | 2 | 1 | 1 | 1 | 8 |
| **Ma2020 [33]** | 0 | 1 | 1 | 1 | 2 | 1 | 1 | 1 | 8 |
| Zhang2020 [34] | 0 | 1 | 1 | 1 | 2 | 1 | 1 | 1 | 8 |
| **Zhao2020 [35]** | 0 | 1 | 1 | 1 | 2 | 1 | 1 | 1 | 8 |
| **Wang2020 [36]** | 0 | 1 | 1 | 1 | 2 | 1 | 1 | 1 | 8 |
| Zhao2021 [37] | 0 | 1 | 1 | 1 | 2 | 1 | 1 | 1 | 8 |
| Zhang2022 [38] | 0 | 1 | 1 | 1 | 2 | 1 | 1 | 1 | 8 |
| Jiao2022 [39] | 0 | 1 | 1 | 1 | 2 | 1 | 1 | 1 | 8 |
| Karadeniz2022 [40] | 0 | 1 | 1 | 1 | 2 | 1 | 1 | 1 | 8 |
| **Guo2022 [41]** | 0 | 1 | 1 | 1 | 2 | 1 | 1 | 1 | 8 |
| **Qin2022 [42]** | 0 | 1 | 1 | 1 | 2 | 1 | 1 | 1 | 8 |
| **Pang2022 [43]** | 0 | 1 | 1 | 1 | 2 | 1 | 1 | 1 | 8 |
| **Shen2023 [44]** | 0 | 1 | 1 | 1 | 2 | 1 | 1 | 1 | 8 |
| **Jin2018a [45]** | 0 | 1 | 1 | 1 | 2 | 1 | 1 | 1 | 8 |
| **Jin2018b [46]** | 0 | 1 | 1 | 1 | 2 | 1 | 1 | 1 | 8 |
| **Neglia2021 [47]** | 0 | 1 | 1 | 1 | 2 | 1 | 1 | 1 | 8 |
| **Yang2021 [48]** | 0 | 1 | 1 | 1 | 2 | 1 | 1 | 1 | 8 |
| **Chen2022 [49]** | 0 | 1 | 1 | 1 | 2 | 1 | 1 | 1 | 8 |
| **Lin2023 [50]** | 0 | 1 | 1 | 1 | 2 | 1 | 1 | 1 | 8 |
| **Gao2021 [51]** | 0 | 1 | 1 | 1 | 2 | 1 | 1 | 1 | 8 |

**Selection**

**1.Representativeness of the exposed cohort;**

**2.Selection of the non exposed cohort;**

**3.Ascertainment of exposure;**

**4.Demonstration that outcome of interest was not present at start of study;**

**Comparability**

**1.Comparability of cohorts on the basis of the design or analysis;**

**Outcome**

**1.Assessment of outcome;**

**2.Was follow-up long enough for outcomes to occur;**

**3.Adequacy of follow up of cohort**

**Table S2. Definition of the primary endpoint.**

| **Study** | **Definition** |
| --- | --- |
| Mao2019 [30] | The composite of cardiac death, non-fatal myocardial infarction, target vessel revascularization, congestive heart failure, and non-fatal stroke. |
| **Luo2019 [31]** | The composite of all-cause death, target vessel revascularization, myocardial infarction during follow-up, unstable angina pectoris requiring hospitalization, heart failure, stroke or transient cerebral ischaemia. |
| **Hu2020 [32]** | The composite of death, non-fatal myocardial infarction, non-fatal stroke or unplanned repeat revascularization. |
| **Ma2020 [33]** | The composite of overall death, non-fatal stroke, non-fatal myocardial infarction, or unplanned repeat revascularization. |
| Zhang2020 [34] | The composite of all-cause death, non-fatal myocardial infarction, revascularization, and cardiac rehospitalization (admission because of angina or heart failure). |
| **Zhao2020 [35]** | The composite of all-cause death, non-fatal myocardial infarction and ischemia-driven revascularization. |
| **Wang2020 [36]** | The composite of all-cause death, non-fatal MI and non-fatal stroke. |
| Zhao2021 [37] | The composite of all-cause death, non-fatal myocardial infarction, non-fatal ischemic stroke, and ischemia-driven revascularization. |
| Zhang2022 [38] | The composite of all-cause death, non-fatal myocardial infarction, nonfatal stroke, revascularization, and cardiac rehospitalization (admission because of angina or heart failure). |
| Jiao2022 [39] | The composite of acute myocardial infarction, coronary artery revascularization and all-cause mortality (cardiac or non-cardiac mortalities). |
| Karadeniz2022 [40] | The composite of all-cause mortality, non-fatal re-infarction, and repeat target vessel revascularization. |
| **Guo2022 [41]** | The composite of cardiac death, non-fatal myocardial infarction, ischemia-driven revascularization, and stroke. |
| **Qin2022 [42]** | The composite of all-cause death, malignant arrhythmia, non-fatal myocardial infarction, target vessel reconstruction, angina pectoris requiring hospitalization, and acute heart failure. |
| **Pang2022 [43]** | The composite of all-cause death, non-fatal myocardial infarction, non-fatal ischemic stroke, and ischemia driven revascularization. |
| **Jin2018a [45]** | The composite of cardiovascular mortality, non-fatal myocardial infarction, stroke, post-discharge revascularization and hospitalized unstable angina. |
| **Jin2018b [46]** | The composite of death, non-fatal myocardial infarction, stroke and post-discharge revascularization. |
| **Yang2021 [48]** | The composite of all-cause mortality, non-fatal myocardial infarction, non-fatal stroke, and target vessel revascularization. |
| **Chen2022 [49]** | The composite of all-cause death, non-fatal myocardial infarction, nonfatal stroke and symptomatic graft failure. |
| **Lin2023 [50]** | The composite of overall death, non-fatal myocardial infarction, and unplanned revascularization. |
| **Gao2021 [51]** | The composite of death, non-fatal myocardial infarction, revascularization, and nonfatal stroke. |

**Table S3. Results of the secondary outcomes.**

| **Secondary outcomes** | **Studies** | **Heterogeneity** | | **Effects model** | **Meta-analysis** | |
| --- | --- | --- | --- | --- | --- | --- |
|  |  | ***P* value** | ***I^2^* value** |  | **HR (95%CI)** | ***P* value** |
| **ACS** | | | | | | |
| **All-cause mortality (Categorized)** | 7 [34-39,44] | 0.52 | 0% | Fixed | 1.43 (1.18-1.73) | 0.0003 |
| **All-cause mortality (Continuous)** | 4 [35,37,39,44] | 0.25 | 27% | Fixed | 1.31 (1.13-1.52) | 0.0003 |
| **Cardiac mortality (Categorized)** | 4 [30,34,38,41] | 0.18 | 39% | Fixed | 1.51 (1.12-2.02) | 0.0006 |
| **Revasculation (Categorized)** | 6 [30,34-38,41] | <0.0001 | 82% | Randomized | 2.42 (1.64-3.57) | <0.00001 |
| **Revasculation (Continuous)** | 2 [35,37] | 0.25 | 23% | Fixed | 2.64 (2.08-3.35) | <0.00001 |
| **Cardiac rehospitalization (Categorized)** | 2 [34,38] | 0.34 | 0% | Fixed | 1.19 (1.03-1.38) | 0.02 |
| **MI (Categorized)** | 7 [30,34-38,41] | 0.65 | 0% | Fixed | 1.58 (1.28-1.96) | <0.0001 |
| **MI (Continuous)** | 2 [35,37] | 0.89 | 0% | Fixed | 3.44 (2.20-5.39) | <0.00001 |
| **Stroke (Categorized)** | 5 [30,36-38,41] | 0.62 | 0% | Fixed | 2.03 (1.39-2.97) | 0.0003 |
| **Stroke (Continuous)** | 1 [35] | - | - | - | 1.86 (0.69-5.04) | 0.22 |
| **CCS** | | | | | | |
| **All-cause mortality (Categorized)** | 3 [47,49,50] | 0.02 | 73% | Randomized | 1.69 (0.52-5.54) | 0.39 |
| **All-cause mortality (Continuous)** | 1 [50] | - | - | - | 1.96 (0.61-6.26) | 0.26 |
| **Revasculation (Categorized)** | 2 [47,50] | 0.71 | 0% | Fixed | 1.30 (1.07-1.59) | 0.01 |
| **Revasculation (Continuous)** | 1 [50] | - | - | - | 1.42 (1.00-2.01) | 0.05 |
| **MI (Categorized)** | 2 [47,50] | 0.77 | 0% | Fixed | 3.80 (1.95-7.40) | <0.0001 |
| **MI (Continuous)** | 1 [50] | - | - | - | 3.18 (1.73-5.83) | 0.0002 |
| **Stroke (Categorized)** | 1 [49] | - | - | - | 2.08 (0.81-5.33) | 0.13 |

**
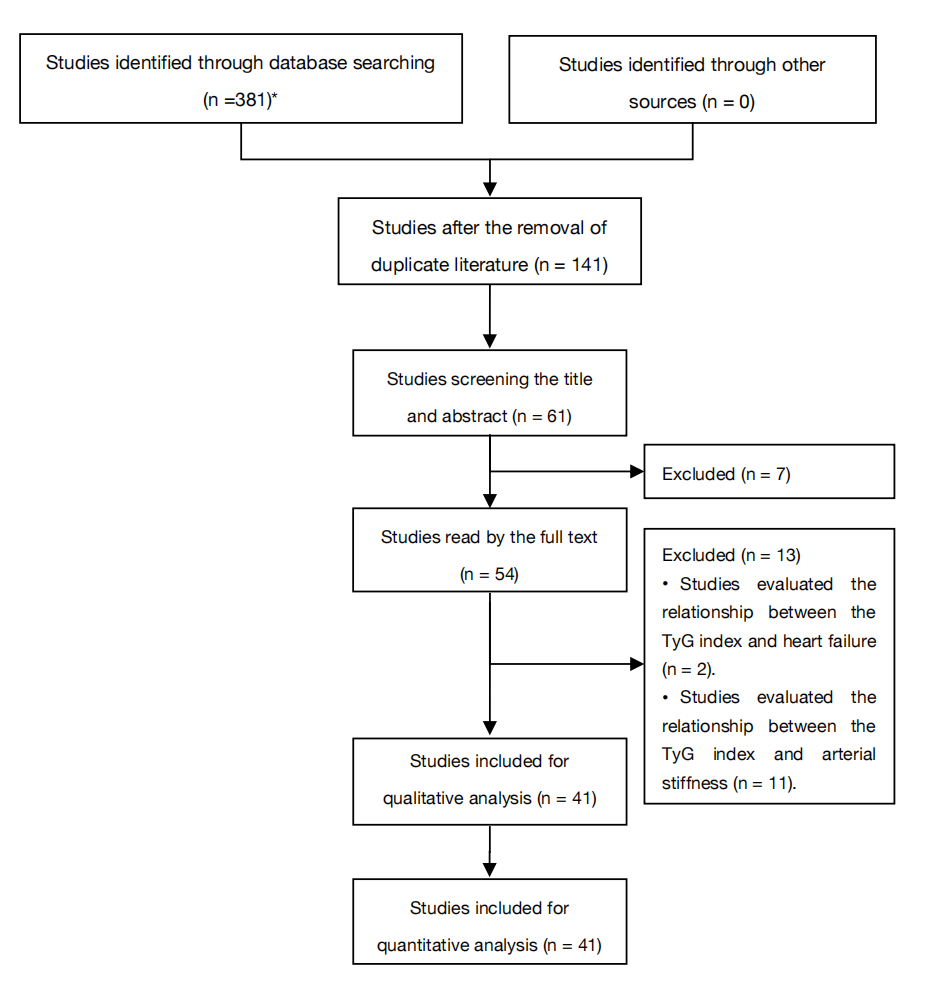
**

**Figure S1. The flow chart of the process (*83 from PubMed, 94 from EMbase, 92 from The Cochrane Library and 112 from Web of Science).**

**
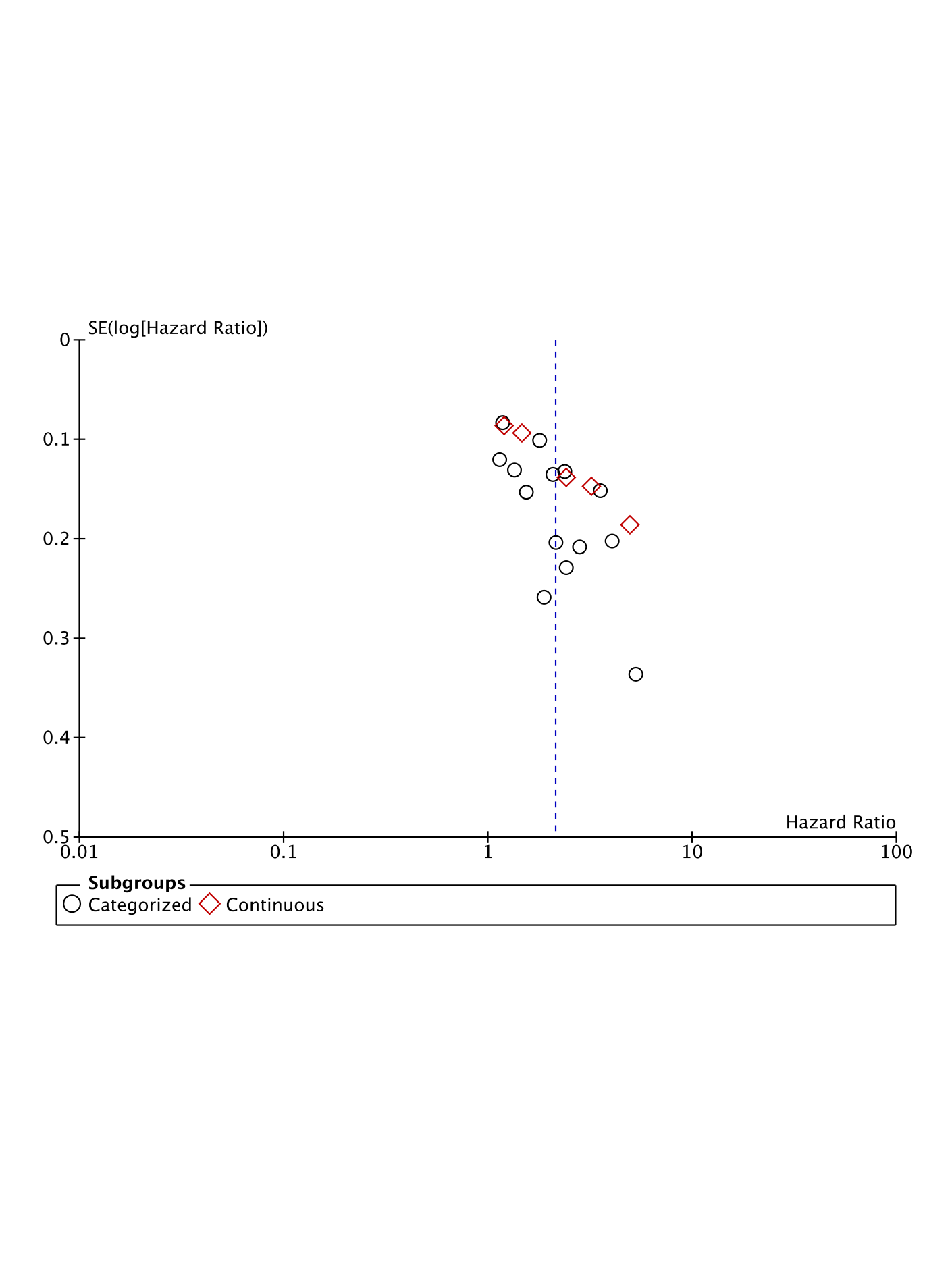
**

**Figure S2. Results of the secondary outcomes.**
